# Supplementary material for: Prevalence, resistance profiles and factors associated with skin and soft-tissue infections at Jinja regional referral hospital: A retrospective study
Source: PLOS Glob Public Health. 2024 Aug 2;4(8):e0003582. doi: 10.1371/journal.pgph.0003582 (PMC11296629; doi:10.1371/journal.pgph.0003582)
Supplement: Attachment — Submitted filename: PGPH-D-24-00292.pdf [file pgph.0003582.s002.pdf]

# PLOS Global Public Health

## Prevalence, resistance profiles and factors associated with skin and soft-tissue infections at Jinja regional referral hospital: A retrospective Study

--Manuscript Draft--

| | |
|---|---|
| **Manuscript Number:** | PGPH-D-24-00292 |
| **Article Type:** | Research Article |
| **Full Title:** | Prevalence, resistance profiles and factors associated with skin and soft-tissue infections at Jinja regional referral hospital: A retrospective Study |
| **Short Title:** | skin and soft-tissue infections and antimicrobial resistance at Jinja hospital |
| **Corresponding Author:** | Fahad Lwigale<br>IDI: Makerere University Infectious Diseases Institute<br>Kampala, Central UGANDA |
| **Order of Authors:** | Fahad Lwigale |
| | Daniel Kibombo |
| | Simon Dembe Kasango |
| | Dickson Tabajjwa |
| | Joseph Kungu |
| | John Bosco Kalule |
| | Morgan Otita |
| | Francis Kakooza |
| | Immaculate Nabukenya |
| | Jonathan Mayito |
| | Innocent B Rwego |
| **Keywords:** | Skin and soft-tissue infections; antimicrobial resistance; hospital-acquired infections; multi-drug resistance; Global Antimicrobial resistance Surveillance System; Jinja |
| **Abstract:** | Skin and soft-tissue infections (SSTI) are common cases of hospital-acquired infections with aetiologic agents exhibiting antimicrobial resistance (AMR). We determined the prevalence of SSTI, proportion of laboratory-investigated cases, AMR-profiles, and factors associated with SSTI and multi-drug resistance (MDR). This study was based on archived data of patients suspected of SSTI from 2019-2021 at Jinja Regional Referral Hospital. The analysis involved 268 randomly selected patient reports using WHONET 2022 and Stata 17 at the 95% confidence level. Prevalence of SSTI was 66.4%. Laboratory-investigated cases were 14.11%. Staphylococcus aureus (n=51) was the most isolated organism. MDR pathogens explained 47% of infections. Methicillin-resistant S. aureus (MRSA) was up to 44%. In addition, 61% of Gram-negatives had the potential to produce extended-spectrum beta-lactamases (ESBL), while 27% were non-susceptible to carbapenems. Ward of admission was significantly associated with infection (aPR=1.78, 95% CI: 1.003-3.18, p-value=0.04). Age category (19-35) was an independent predictor for MDR infections (aPR=2.30, 95%CI:1.02-5.23, p-value=0.04). The prevalence is relatively high with MDR pathogens responsible for almost half of the infections. Routine use of culture and sensitivity testing should be done for proper infection management. Gentamicin and ciprofloxacin can be considered for empirical management of emergency SSTI suspected of S. aureus. Recognizing SSTI under the Global Antimicrobial resistance Surveillance System (GLASS) would lead to improved preparedness and response to AMR. |
| **Additional Information:** | |
| **Question** | **Response** |
| **Financial Disclosure** | The author(s) received no specific funding for this work. |

*Powered by Editorial Manager® and ProduXion Manager® from Aries Systems Corporation*

| | |
|---|---|
| Enter a financial disclosure statement that describes the sources of funding for the work included in this submission and the role the funder(s) played. This includes grants and any commercial funding of the work or authors.<br><br>This statement will be typeset if the manuscript is accepted for publication.<br><br>*Please review the submission guidelines and the instructions link below for detailed requirements and guidance.* | |
| **Competing Interests**<br><br>On behalf of all authors, disclose any competing interests that could be perceived to bias this work.<br><br>This statement will be typeset if the manuscript is accepted for publication.<br><br>*Please review the instructions link below and PLOS Global Public Health's competing interests policy to determine what information must be disclosed at submission.* | The authors have declared that no competing interests exist. |
| **Data Availability**<br><br>Before publication, Authors are required to make fully available and without restriction all data underlying their findings. Please see our PLOS Data Policy page for detailed information on this policy.<br><br>A **Data Availability Statement**, detailing where the data can be accessed, is required at first submission. Insert your Data Availability Statement in the box below. | The dataset is published and readily accessible via https://doi.org/10.5061/dryad.rjdfn2zkh |

| | |
|---|---|
| Please see the data reporting section of our submission guidelines for instructions on what you need to include in your Data Availability Statement.<br><br>This statement will be typeset if the manuscript is accepted for publication.<br><br>*PLOS allows rare exemptions to address legal and ethical concerns. If you have legal or ethical restrictions, please use the box below to detail these in full sentences for the Journal team to consider.* | |

*Powered by Editorial Manager® and ProduXion Manager® from Aries Systems Corporation*

Manuscript Click here to access/download;Manuscript;Manuscript.docx

# Prevalence, resistance profiles and factors associated with skin and soft-tissue infections at Jinja regional referral hospital: A retrospective Study

Fahad Lwigale[1,2*], Daniel Kibombo[1], Simon Dembe Kasango[1,3], Dickson Tabajjwa[1], Joseph Kungu[2], John Bosco Kalule[2], Morgan Otita[1], Francis Kakooza[1], Immaculate Nabukenya[1,2], Jonathan Mayito[1], Innocent B. Rwego[2]

[1]Global Health Security Program, Infectious Diseases Institute, College of Health Sciences, Makerere University, Kampala, Uganda

[2]School of Biosecurity, Biotechnical and Laboratory Sciences, College of Veterinary Medicine Animal Resources and Biosecurity, Makerere University, Kampala, Uganda

[3]Uganda National Health Research Organization, Ministry of Health, Kampala, Uganda

[*]Correspondence

Email: fahadlwigale@gmail.com (FL)

## Abstract:

Skin and soft-tissue infections (SSTI) are common cases of hospital-acquired infections with aetiologic agents exhibiting antimicrobial resistance (AMR). We determined the prevalence of SSTI, proportion of laboratory-investigated cases, AMR-profiles, and factors associated with SSTI and multi-drug resistance (MDR). This study was based on archived data of patients suspected of SSTI from 2019-2021 at Jinja Regional Referral Hospital. The analysis involved 268 randomly selected patient reports using WHONET 2022 and Stata 17 at the 95% confidence level. Prevalence of SSTI was 66.4%. Laboratory-investigated cases were 14.11%. *Staphylococcus aureus* (n=51) was the most isolated organism. MDR pathogens explained 47% of infections. Methicillin-resistant *S. aureus* (MRSA) was up to 44%. In addition, 61% of Gram-negatives had the potential to produce extended-spectrum beta-lactamases (ESBL), while 27% were non-susceptible to carbapenems. Ward of admission was significantly associated with infection (aPR=1.78, 95% CI: 1.003-3.18, p-value=0.04). Age category (19-35) was an independent predictor for MDR infections (aPR=2.30, 95%CI:1.02-5.23, p-value=0.04). The prevalence is relatively high with MDR pathogens responsible for almost half of the infections. Routine use of culture and sensitivity testing should be done for proper infection management. Gentamicin and ciprofloxacin can be considered for empirical management of emergency SSTI suspected of *S. aureus*. Recognizing SSTI under the Global Antimicrobial resistance Surveillance System (GLASS) would lead to improved preparedness and response to AMR.

# Introduction

Antimicrobial resistance (AMR) is an emerging public health threat of concern globally (1, 2). It has been noted to be responsible for negative social, economic, and health consequences; higher healthcare costs; increased Disability Adjusted Life Years (DALYs); and decreased economic growth (3, 4). This burden is projected to increase in the near future if no proper attention is paid to managing it (3). Improper use of antimicrobials is one of the main factors contributing to the development of AMR (1, 5, 6). This has been reported in Uganda, which includes the prescription of antimicrobial agents for the wrong condition in lower health facilities (1, 5, 7). Using microbiology services such as culture and sensitivity (C&S) testing enables determination of the identity of the causative agents and the appropriate antimicrobial agents that can be used for proper management of individuals with infections, such as skin and soft-tissue infections (SSTI)(8, 9). However, this this is still far from the practice in this setting as most clinical management is still empirical. Laboratory surveillance is conducted in Uganda as part of the mechanisms to tackle AMR in line with the global action plan for AMR (10).

Skin and soft-tissue infections are some of the most commonly encountered cases of hospital-acquired infections (HAI) and are characterized by AMR mainly among post-operative patients in low and middle-income countries (LMICs) (11-13). These are a type of infection involving colonization and inflammation of the epidermis, dermis, and subcutaneous tissues (9, 14). The colonizing agents such as bacteria are commonly from the hospital environment such as sinks, surgical beds, staff and wound dressings. These have been reported to be highly resistant to the commonly used antimicrobial agents (15-17). Various factors have been reported to influence the occurrence of SSTI including the presence of comorbidities, 48-hour duration of surgical

antimicrobial prophylaxis, contaminated and dirty wounds, sex, lack of prophylactic antibiotic treatment, postoperative length of stay, American Society of Anesthesiologists (ASA) score of ≥ 2, and the timing of prophylactic antibiotic, which is more beneficial 1-2 hours before surgery (13, 18, 19). Other independent factors include open surgery, emergency operations, male sex, long duration of surgery, intraoperative blood transfusion, age, smoking, and HIV (14, 20, 21).

Surgical site infections prevalence was between 1.5% and 3.64% in China (22, 23), 11.7% in Malaysia (19), 9.85% in parts of Europe (24), and ranged from 10.3% to 15.6% in sub-Saharan Africa (25). Microbial growth was 68.5% in Pakistan (6), 16.3% in Saudi Arabia (20), 62.1% in Sierra Leone (21), and 70.0% in Ethiopia(26). Positivity rates ranged from 81.93% to 92% in Uganda (27-29). Gram-negative organisms, such *as Pseudomonas aeruginosa, Acinetobacter species*, *Klebsiella species, Escherichia coli*, *Proteus species*, and other *coliforms* are the ones mostly responsible for wound infections (6, 21, 30). However, *Staphylococcus aureus* has been observed as the dominant wound pathogen elsewhere (2, 31-33). Mixed infections have also been reported, where the infection is due to more than one etiologic agent (12, 31, 34). A significant number of the isolated bacteria are multi-drug resistant (MDR) (21, 28, 34). The *Enterobacterales* were collectively resistant to ampicillin while the overall *S. aureus* resistance profile was oxacillin (23.9%), ciprofloxacin (18.9%), trimethoprim/sulfamethoxazole (55.7%) and clindamycin (8.8%) (2). Multi-drug resistant isolates were up to 22.6% in Poland(33) while MRSA levels have been reported up to 30.3% in Kuwait (34). The least resistance was observed against gentamicin (24%) and ciprofloxacin (27%) (26).

There is insufficient utilization of microbiology laboratory services during infection management in some health facilities in Uganda (5, 27), leaving room for non-targeted therapy. Further, the

Global Antimicrobial Resistance and antimicrobial use Surveillance System (GLASS) (10, 35) does not currently consider SSTI despite their high cultural yields and associated AMR observed in the microbiology laboratory (26-28). This limits the availability of some essential information for an appropriate response towards the AMR epidemic. The actual status of SSTI is unknown at Jinja Regional Referral Hospital (RRH), with little knowledge about their influencers. This study therefore determined the prevalence of SSTI, proportion of SSTI that undergo laboratory investigation, common causative organisms and their antibiotic resistance profiles, and factors associated with SSTI and MDR infections. This information would enable formulation or/and review of guidelines for better management of SSTI and improve regional antimicrobial stewardship practices for containment of AMR.

# Materials and methods

## Study design

This was a retrospective study based on the abstraction of socio-demographic and clinical charts of patients diagnosed with SSTI from January 2019 to December 2021. The data was accessed for analysis in June 2023. The study took place shortly after the establishment of microbiology services, majorly culture and sensitivity testing in 2018 and was made readily available for routine use in the region.

## Study setting

The study was carried out at Jinja RRH in the Eastern-central region of Uganda (**Fig 1**). The hospital is located in the center of Jinja city. Jinja is a focal point and refreshment area along the

path from the Ugandan capital, Kampala towards the Kenyan border. This is a path that has encountered several traffic accidents in recent years, the majority of whose victims are managed at Jinja RRH(36-38). This facility serves the Eastern-central region of Uganda which involves a population of approximately 4.5 million people from within Jinja and the surrounding areas such as Iganga, Mayuge, Bugiri, Kamuli, Buikwe, Lugazi, Kayunga, and Mukono districts among others (39). The facility is equipped with a laboratory accredited by the South African National Accreditation System based on the requirements of ISO 15189 (40).

**Fig 1. A map showing the location of the study site.**

## Laboratory procedures

The laboratory data was previously generated using conventional microbiology methods for bacterial identification and Antimicrobial Susceptibility Testing(AST) was carried out using the Kirby-Bauer disk-diffusion method following the Clinical and Laboratory Standards Institute (CLSI) guidelines (41-43). The laboratory observes quality control internally and by engagement in routine external quality assessments. Isolated organisms are periodically used for inter-laboratory comparison with the reference laboratory.

## Study Population and Sampling

The investigated population included records of patients who were managed for SSTI with or without laboratory testing. Records for both inpatients and outpatients were considered. Laboratory records without updated results were not included. Only the first isolate of any patient was considered for analysis to generate antimicrobial resistance profiles.

Out of 526 laboratory patient records, a total of 268 reports were selected by systematic random sampling for the study. The sample size was calculated using the formula ($n =p(1-p)z^2/d^2$) and had a power of 80%  to estimate the prevalence and factors associated with SSTI at the 95% confidence level.

# Data collection

## Extraction of Laboratory generated data

The data sample frame with the necessary variables for the study was accessed on June, 30th 2023 and extracted from the electronic African Laboratory Information System (ALIS) of Jinja RRH laboratory into an Excel sheet. This included demographic and clinical data of patients who underwent laboratory testing including age, sex, ward, hospitalization history before testing, isolated organism, and antimicrobial susceptibility testing (AST) results where applicabl[illegible]

## Review of patients’ files

In addition to the existing laboratory-generated data, patients' files for the same study patients were sought and examined for more data necessary to investigate the associated factors. This was obtained using a predesigned data extraction tool transformed into the kobo-collect mobile application with kobo-toolbox open access software. The tool was piloted to confirm functionality

and ability to obtain the required data before the actual study. This involved the entry of data for ten random patients admitted to the surgical ward into the electronic tool. This was saved on an online server and the aggregated data was downloadable in the form of a spreadsheet. Research assistants including a nurse, and records personnel were trained on the research tool in the same period and they became familiar with the data collection process. The collected data included admission periods, history of undergoing surgery, type of surgery, the theatre involved, and whether a patient was treated based on culture and sensitivity results from the laboratory.

## Data Extraction from the District Health Information System (DHIS)

The overall number of patients diagnosed and treated for SSTI in the study period was obtained to aid the determination of the proportion of suspected SSTI that underwent microbiology testing for confirmation. This was done by examining standard Health Management Information System (HMIS 108 and HMIS 105:01) reports from the electronic DHIS2. The medical conditions considered for counting as part of SSTI included infections affecting the SSTI such as the middle ear, gangrenes, skin abscesses, and similar ones whether acute or chronic. These included the following as stated in the HMIS tools. Skin diseases (CD14), tetanus (CD15), otitis media (EN01), otitis externa (EN10), and burn injuries (OT04) from HMIS 105:01. Those in HMIS 108 included musculoskeletal and connective tissue diseases (LD04), cutaneous ulcers (LD09), osteomyelitis (CD11), tetanus (CD13), rheumatoid arthritis (RM01), septic arthritis (RM02), osteoarthritis (RM03), otitis media (EN01), injuries (IN01), diseases of the skin (LD03) and sepsis related to pregnancy (MC07). The total sum of diagnoses from the stated conditions was treated as the total number of patients with indication and treated for SSTI in the study period.

# Ethics Statement

The Jinja Hospital Research and Ethics Committee approved the study with registration number **JREC 395/2023** and granted a waiver of consent.

# Data Analysis

The data collected was entered and cleaned in Microsoft Excel. Statistical analysis was performed using Stata 17. Categorical variables were summarized in the form of frequencies and percentages and presented by bar graphs and tables. Continuous variables were presented as means with standard deviation(SD). Prevalence of SSTI was calculated overall and relative to wards of admission. Microbiology service utilization to confirm and manage suspected SSTI was estimated using two proportions; 1) Percentage of suspected SSTI investigated by culture and sensitivity (C&S) was obtained as a proportion of C&S tests done to the total sum of SSTI diagnoses ($\sum$C&S tests $\div$ $\sum$SSTI Indications); 2) Percentage of patients managed based on microbiology (C&S) test results were calculated as the number of patients with de-escalation in treatment with antibiotics basing on C&S divided by the number of patients with positive C&S test ($\sum$Patients with de-escalation $\div$ $\sum$ Positive C&S tests). WHONET 2022 was used for antimicrobial susceptibility data analysis. Organisms with a minimum number of 30 isolates were considered individually to generate antimicrobial susceptibility profiles. Otherwise, organisms were grouped based on their microbiologic characteristics such as the order, Enterobacterales, and antibiograms generated for the group. Multi-drug resistance (MDR) was defined as an isolate resistant to at least three antibiotics of different clinical categories (44). Bivariate analysis was performed to test for associations between the presence of SSTI and MDR etiology independently with possible

predictors. Factors with a P-value <0.2 were followed up with multivariate analysis by modified Poisson regression. Statistical significance was defined as a P-value of <0.05 at the 95% confidence level.

# Results

## Demographic Characteristics

A total of 268 patient reports were included in the study. Of these, 55% (n = 148) belonged to males. The patients had a mean age of 31 years (SD = 20.8) and most of the patients (31%, n = 84) belonged to the age group of 19-35 years followed by 36-59 years (28%, n = 76). Nearly a third of the patients (n = 88) were admitted to the surgical unit. Moreover, two-thirds of the study patients were undergoing antibiotic treatment before any microbiology testing was done (**Table 1).**

**Table 1. Demographic characteristics of the study population**

| *Variable* | | *n* | *(%)* |
|---|---|---|---|
| *Year of Case* | 2019 | 44 | 16.4 |
| | 2020 | 120 | 44.8 |
| | 2021 | 104 | 38.8 |
| *Sex* | Male | 148 | 55.2 |
| | Female | 120 | 44.8 |

| | | | |
|---|---|---|---|
| *Age Category* | ≤12 | 48 | 17.9 |
| | 13-18 | 31 | 11.6 |
| | 19-35 | 84 | 31.3 |
| | 36-59 | 76 | 28.4 |
| | ≥60 | 29 | 10.8 |
| *Ward/Department* | Accidents and emergency | 16 | 6.0 |
| | Gynecology | 13 | 4.9 |
| | Maternity | 15 | 5.6 |
| | Medical | 15 | 5.6 |
| | Surgical | 88 | 33 |
| | Outpatient Department | 38 | 14.2 |
| | Orthopedics | 34 | 12.7 |
| | Private wing | 8 | 3.0 |
| | Pediatrics | 10 | 3.7 |
| | Others | 31 | 11.6 |
| | Unknown | 51 | 19 |

| | | | |
|---|---|---|---|
| *On antibiotics before testing* | Yes | 176 | 65.7 |
| | No | 92 | 34.3 |
| *Undergoing surgery* | Yes | 11 | 4.1 |
| | No | 98 | 36.6 |
| | Unknown | 159 | 59.3 |

## Prevalence of Skin and Soft Tissue Infections

The prevalence of SSTI was 66.4% ([illegible] = 178, 95% CI = 60.7-72.1). Among the cases, 56.7% (n = 101) were males. The most affected age groups were those between 19 and 35 years (29.8%) followed by 36-59(27.5%). Polymicrobial growth was observed in 7.2% (n=18) of the cases. Of these, *Candida species* 2% (*n*=5) were the major co-infection.

The pediatric ward had the highest prevalence of SSTI of up to 80% (8/10) while the accidents and emergency unit had the lowest, 43.7% (7/ 16) (**Table 2**).

**Table 2. Prevalence of SSTI in the Respective Wards of Admission.**

| *Ward/Department* | *Total* no. of *Patients* | *Infection Frequency* | *Percentage* |
|---|---|---|---|
| *Pediatrics* | 10 | 8 | 80 |

| | | | |
|---|---|---|---|
| *Medical* | 15 | 8 | 53 |
| *Accidents and emergency* | 16 | 7 | 43.7 |
| *Surgical* | 88 | 66 | 75 |
| *Outpatient* | 38 | 25 | 65.7 |
| *Maternity* | 15 | 8 | 53 |
| *Orthopedics* | 34 | 20 | 58.8 |
| *Private* | 8 | 6 | 75 |
| *Antenatal* | 13 | 7 | 53.8 |
| *Others* | 31 | 23 | 74.2 |

Approximately 3,720 cases were diagnosed and treated for SSTI during the study period. Only 14.1% (n = 526) of C&S tests were done among patients suspected of SSTI. In 2019, there were 8.1% (104/1278) cases that underwent laboratory testing for SSTI. Meanwhile, years 2020 and 2021 respectively had 21.1% (226/1072) and 14.3% (196/1370) cases diagnosed by the laboratory to guide management (**Fig 2**).

Only 2.1% (n = 11) of the patients' records were found to have C&S test results in patient files. The treatment records for outpatients were not readily available. Therefore, the representative percentage of de-escalation based on the C&S report could not be estimated.

**Fig 2. Proportion of cases that underwent laboratory investigation.** Less than 30% of cases involved laboratory testing in all the year periods studied.

# Antimicrobial resistance patterns of selected bacteria responsible for skin and soft-tissue infections

## Major bacteria responsible for the observed SSTI

The observed organism growth yielded 2[illegible] isolates (**Fig 3**). Approximately 44.4% (n = 119) of the infections were due to Gram-negative bacteria, 19.4% (n = 52) were Gram-positive cocci and 2.6% (n = 7) were yeasts. Most of the infections were due to *S. aureus* (19%, n = 51) followed by *E. coli* (10.4%, n = 28), *Colifor*[illegible] (10.4%, n = 28), *Klebsiella species* (6.7%, n = 18), *Citrobacter species* (6.3%, n = 17), and *Proteus species* 3.3%, n = 9). Non-*Enterobacterales* were mainly made up of *Pseudomonas species* (4.1%, n = 11) and *Acinetobacter species* (2.6%, n = 7). Nearly 3% (n = 7) of isolates from the SSTI were *Candida species*. Bacterial isolates also included *Coagulase Negative Staphylococci* (*CoNS*)(n=22).

**Fig 3. Frequency distribution of the isolated organisms responsible for the SSTI.** Most isolates were S. aureus.

## Percentage resistance of the bacteria to common antibiotics

Up to 47% (n = 79) of the infections were due to MDR pathogens. Among the Gram-negative bacteria, 61.3% (n = 73) were resistant to third-generation cephalosporins and hence possible ESBL producers while 27.7% (n = 33) were non-susceptible to carbapenems. All the tested isolates for *S. aureus* were resistant to penicillin G (n = 23) (**Fig 4**). Over 44.4% (n = 8, 95% C.I: 22.4-68.7) of the tested isolates were methicillin-resistant *Staphylococcus aureus* (MRSA; **Table 3**).

The highest percentage resistance among *enterobacterales* altogether was against ampicillin (97.1%, n=34). This group was least resistant to meropenem (0%) and imipenem (15.6%) (**Table 4**).

**Fig 4. Percentage resistance for *S. aureus*.** The highest level of resistance was observed to be against penicillin G.

**Table 3. Antimicrobial susceptibility profile for Staphylococcus aureus.**

| *Antibiotic name* | *Antibiotic class* | *Breakpoints* | *Number Tested* | *%R* | *%I* | *%S* | *%R, 95%C.I.* | *%S, 95%C.I.* |
|---|---|---|---|---|---|---|---|---|
| **Cefoxitin* | Cephems | S >= 22 | 18 | 44.4 | 0.0 | 55.6 | 22.4-68.7 | 31.3-77.6 |
| *Chloramphenicol* | Phenicols | 13 - 17 | 16 | 6.3 | 12.5 | 81.3 | 0.3-32.3 | 53.7-95.0 |
| *Ciprofloxacin* | Quinolones | 16 - 20 | 48 | 41.7 | 16.7 | 41.7 | 27.9-56.7 | 27.9-56.7 |
| *Clindamycin* | Lincosamides | 15 - 20 | 36 | 36.1 | 16.7 | 47.2 | 21.3-53.8 | 30.8-64.3 |
| *Erythromycin* | Macrolides | 14 - 22 | 37 | 70.3 | 21.6 | 8.1 | 52.8-83.6 | 2.1-23.0 |
| *Gentamicin* | Aminoglycosides | 13 - 14 | 35 | 28.6 | 8.6 | 62.9 | 15.2-46.5 | 44.9-78.0 |
| *Penicillin G* | Penicillins | S >= 29 | 23 | 100.0 | 0.0 | 0.0 | 82.2-100 | 0.0-17.8 |
| *Tetracycline* | Tetracyclines | 15 - 18 | 9 | 22.2 | 44.4 | 33.3 | 3.9-59.8 | 9.0-69.1 |

| | | | | | | | | |
|---|---|---|---|---|---|---|---|---|
| *Trimethoprim/Sulfamethoxazole* | Folate pathway inhibitors | 11 - 15 | 7 | 100.0 | 0.0 | 0.0 | 56.1-100 | 0.0-43.9 |

R-Resistant; S-Susceptible; I-Intermediate; C.I-Confidence Interval

*Note: Cefoxitin is the recommended surrogate test agent for determining the susceptibility of *S. aureus* to Oxacillin or Methicillin using the disk-diffusion method (41-43). *S. aureus* isolates that are resistant to Cefoxitin are regarded as Methicillin Resistant *S. aureus* (MRSA).

**Table 4. Antimicrobial susceptibility profile for Enterobacterales.**

| *Antibiotic name* | *Antibiotic class* | *Breakpoints* | *Number Tested* | *%R* | *%I* | *%S* | *%R, 95%C.I.* | *%S,95%C.I.* |
|---|---|---|---|---|---|---|---|---|
| *Amikacin* | Aminoglycosides | 15 - 16 | 19 | 15.8 | 26.3 | 57.9 | 4.2-40.5 | 34.0-78.9 |
| *Amoxicillin/Clavulanic acid* | Beta-lactam+Inhibitors | 14 - 17 | 7 | 71.4 | 0.0 | 28.6 | 30.3-94.9 | 5.1-69.7 |
| *Ampicillin* | Penicillins | 14 - 16 | 35 | 97.1 | 2.9 | 0.0 | 83.4-99.9 | 0.0-12.3 |
| *Cefotaxime* | Cephalosporin III | 23 - 25 | 18 | 77.8 | 16.7 | 5.6 | 51.9-92.6 | 0.3-29.4 |

| | | | | | | | | |
|---|---|---|---|---|---|---|---|---|
| *Ceftazidime* | Cephalosporin III | 18 - 20 | 38 | 73.7 | 10.5 | 15.8 | 56.6-86.0 | 6.6-31.9 |
| *Cefuroxime* | Cephalosporin II | 15 - 17 | 10 | 80.0 | 0.0 | 20.0 | 44.2-96.5 | 3.5-55.8 |
| *Chloramphenicol* | Phenicols | 13 - 17 | 78 | 46.2 | 10.3 | 43.6 | 34.9-57.8 | 32.6-55.3 |
| *Ciprofloxacin* | Fluoroquinolone | 22 - 25 | 83 | 51.8 | 9.6 | 38.6 | 40.6-62.8 | 28.3-49.9 |
| *Gentamicin* | Aminoglycosides | 13 - 14 | 71 | 33.8 | 12.7 | 53.5 | 23.3-46.1 | 41.4-65.3 |
| *Imipenem* | Carbapenems | 20 - 22 | 45 | 15.6 | 8.9 | 75.6 | 7.0-30.1 | 60.1-86.6 |
| *Meropenem* | Carbapenems | 20 - 22 | 5 | 0.0 | 0.0 | 100.0 | 0.0-53.7 | 46.3-100 |
| *Tetracycline* | Tetracyclines | 12 - 14 | 18 | 72.2 | 0.0 | 27.8 | 46.4-89.3 | 10.7-53.6 |
| *Trimethoprim/Sulfamethoxazole* | Folate pathway inhibitors | 11 - 15 | 21 | 90.5 | 4.8 | 4.8 | 68.2-98.3 | 0.2-25.9 |

R-Resistant; S-Susceptible; I-Intermediate; C.I-Confidence Interval

Non-*enterobacterales* composed of *Pseudomonas* and *Acinetobacter* species together (n=18) had a percentage resistance of 55.6% (n = 7) for piperacillin, 50% (n = 1) for amikacin, 50% (n = 7) for ceftazidime, 36.4% (n = 5) for ciprofloxacin, 25% (n = 2) for gentamicin, and 22.2% (n = 2) for Imipenem (**Fig 5**).

**Fig 5. Percentage resistance for Non-enterobacterales.** This group involved Pseudomonas species and Acinetobacter species combined for analysis.

## Factors associated with SSTI

Patients in the surgical ward were significantly more likely to develop an SSTI compared to those in the A&E ward (aPR = 1.78, 95%, CI:1.003-3.18, p = 0.04). Age, gender, hospital admission hours, and status of antibiotic use before testing were not independently associated with risk for SSTI. (**Table 5**).

**Table 5. Factors Associated with Skin and Soft Tissue Infections.**

| ***Variable*** | **Infection** | | **Bivariate Analysis** | **Multivariate Analysis** |
|---|---|---|---|---|
| *Ward, n (%)* | **No** (n=90) | **Yes** (n=178) | **cPR**(95%CI),p-value | **aPR**(95%CI), p-value |
| *A&E* | 9(10.0) | 7(3.9) | 1.00 | 1.00 |
| *Gynecology* | 6(6.7) | 7(3.9) | 1.23(0.58-2.61) 0.58 | 1.32 (0.61-2.88) 0.48 |

| | | | | |
|---|---|---|---|---|
| *Maternity* | 7(7.8) | 8(4.5) | 1.22(0.59-2.53)0.59 | 1.30 (0.61-2.77) 0.48 |
| *Medical* | 7(7.8) | 8(4.5) | 1.22(0.59-2.53) 0.59 | 1.28 (0.61-2.70) 0.51 |
| *OPD* | 13(14.4) | 25(14.0) | 1.50(0.82-2.75) 0.18 | 1.60 (0.86-2.98) 0.13 |
| *Orthopedics* | 14(15.6) | 20(11.2) | 1.34(0.72-2.51) 0.35 | 1.43 (0.76-2.69) 0.26 |
| *Other* | 8(8.9) | 23(12.9) | 1.69 (0.94-3.07) 0.08 | 1.81 (0.98-3.35) 0.05 |
| *Private* | 2(2.2) | 6(3.4) | 1.71(0.86-3.40) 0.12 | 1.86 (0.91-3.77) 0.08 |
| *Pediatrics* | 2(2.2) | 8(4.5) | 1.83(0.97-3.46) 0.06 | 1.78 (0.92-3.46) 0.08 |
| *Surgical* | 22(24.4) | 66(37.1) | 1.71(0.97-3.03) 0.06 | 1.78 (1.00-3.18) **0.04** |
| *Sex, n (%)* | | | | |
| *Female* | 43(47.8) | 77(43.3) | 1.00 | 1.00 |
| *Male* | 47(52.2) | 101(56.7) | 1.06(0.89-1.26) 0.48 | 1.04 (0.86-1.24) 0.702 |
| *Age Group, n (%)* | | | | |
| *12&below yrs.* | 14(15.6) | 34(19.1) | 1.00 | 1.00 |
| *13-18yrs* | 10(11.2) | 21(11.8) | 0.95(0.70-1.29) 0.77 | 0.97 (0.70-1.33) 0.841 |
| *19-59yrs* | 31(34.4) | 53(29.8) | 0.89(0.69-1.13) 0.35 | 0.90 (0.68-1.19) 0.479 |
| *60+yrs* | 35(38.9) | 70(39.3) | 0.94(0.75-1.18) 0.60 | 0.92 (0.71-1.19) 0.534 |

| | | | | |
|---|---|---|---|---|
| *Undergoing surgery, n (%)* | | | | |
| *No* | 85(94.4) | 172(96.6) | 1.00 | |
| *Yes* | 5(5.6) | 6(3.6) | 0.81(0.47-1.40) 0.46 | |
| *Surgical type, n (%)* | | | | |
| *Elective* | 2(2.2) | 3(1.7) | 1.00 | 1.00 |
| *Others* | 88 (97.7) | 175 (98.3) | 1.10 (0.54-2.28) 0.77 | 0.52 (0.20-1.30) 0.162 |
| *Hospital admission >48hrs, n (%)* | | | | |
| *No* | 49(54.4) | 100 (56.2) | 1.00 | 1.00 |
| *Yes* | 41(45.6) | 78(43.8) | 0.97(0.82-1.16) 0.78 | 1.004 (0.79-1.27) 0.974 |
| *Year of case* | | | | |
| *2019* | 13 (14.4) | 31 (17.4) | 1.00 | 1.00 |
| *2020* | 45 (50) | 75 (42.1) | 0.89 (0.70-1.12) 0.32 | 0.96 (0.73-1.27) 0.790 |
| *2021* | 32 (35.6) | 72 (40.5) | 0.98 (0.78-1.23) 0.88 | 1.05 (0.80-1.37) 0.732 |

| | | | | |
|---|---|---|---|---|
| *Antibiotic use before testing* | | | | |
| *No* | 31 (34.4) | 61 (34.3) | 1.00 | 1.00 |
| *Yes* | 59 (65.6) | 117 (65.7) | 1.002 (0.84-1.20) 0.97 | 1.04 (0.82-1.32) 0.721 |
| *Type of theatre* | | | | |
| *Others* | 86 (95.6) | 172 (96.6) | 1.00 | |
| *Main theatre* | 4 (4.4) | 6 (3.4) | 1.11 (0.66-1.86) 0.68 | |

## Factors associated with MDR pathogens responsible for SSTI.

Age was the only factor significantly associated with MDR, where patients aged 19 to 59 years were over two times more likely to have MDR pathogens compared to those who were 12 years or younger (aPR = 2.30, 95%CI:1.02-5.23, p = 0.04) (**Table 6**).

**Table 6. Factors associated with MDR pathogens among patients with Skin and Soft Tissue Infections.**

| ***Variable*** | **MDR** | | **Bivariate Analysis** | **Multivariate Analysis** |
|---|---|---|---|---|
| *Ward, n (%)* | **No** (n=189) | **Yes** (n=79) | **cPR**(95%CI), p-value | **aPR**(95%CI), p-value |
| *A&E* | 12(6.4) | 4(5.1) | 1.00 | 1.00 |

| | | | | |
|---|---|---|---|---|
| *gynecology* | 8(4.2) | 5(6.3) | 1.54(0.51-4.60) 0.44 | 1.21 (0.39-3.74) 0.742 |
| *maternity* | 11(5.8) | 4(5.1) | 1.07(0.32-3.53)0.91 | 0.88 (0.25-3.09) 0.842 |
| *medical* | 12(6.4) | 3(3.8) | 0.80(0.21-3.00) 0.74 | 0.71 (0.19-2.67) 0.616 |
| *OPD* | 28(14.8) | 10(12.7) | 1.05(0.39-2.87)0.92 | 1.05 (0.39-2.80) 0.917 |
| *Orthopedics* | 25(13.2) | 9(11.4) | 1.06(0.38-2.93) 0.91 | 0.84 (0.29-2.41) 0.739 |
| *Other* | 22(11.6) | 9(11.4) | 1.16(0.42-3.20) 0.77 | 1.50 (0.54-4.15) 0.432 |
| *Private* | 5(2.7) | 3(3.8) | 1.50(0.44-5.16) 0.52 | 1.49 (0.45-4.95) 0.512 |
| *Pediatrics* | 8(4.2) | 2(2.5) | 0.80(0.18-3.60) 0.77 | 1.49 (0.29-7.39) 0.627 |
| *Surgical* | 58(30.7) | 30(38.0) | 1.36 (0.56-3.35) 0.49 | 1.18 (0.47-2.94) 0.719 |
| *Sex, n (%)* | | | | |
| *Female* | 88(46.6) | 32(40.5) | 1.00 | 1.00 |
| *Male* | 101(53.4) | 47(59.5) | 1.19(0.81-1.74) 0.36 | 1.25 (0.84-1.88) 0.276 |
| *Age, n (%)* | | | | |
| *12&below yrs.* | 40(21.2) | 8(10.1) | 1.00 | 1.00 |
| *13-18yrs* | 22(11.6) | 9(11.4) | 1.74(0.75-4.03) 0.19 | 2.14 (0.79-5.73) 0.13 |
| *19-59yrs* | 54(28.6) | 30(38.0) | 2.14 (1.06-4.29) 0.03 | 2.30 (1.02-5.23) **0.04** |
| *60+yrs* | 73(38.6) | 32(40.5) | 1.82(0.91-3.67) 0.09 | 1.96 (0.87-4.12) 0.10 |

| | | | | |
|---|---|---|---|---|
| *Undergoing surgery, n (%)* | | | | |
| *No* | 181(95.8) | 76(96.2) | 1.00 | |
| *Yes* | 8(4.2) | 3(3.8) | 0.92(0.34-2.46) 0.87 | |
| *Surgical type, n (%)* | | | | |
| *Elective* | 2(1.1) | 3(3.8) | 3.69 (0.60-22.52) 0.15 | |
| *Others* | 187 (98.9) | 76(96.2) | 1.00 | |
| *Hospital admission >48hrs, n (%)* | | | | |
| *No* | 112(59.3) | 37(46.8) | 1.00 | 1.00 |
| *Yes* | 77(40.8) | 42(53.2) | 1.42(0.98-2.05) 0.063 | 1.17 (0.71-1.94) 0.541 |
| *Year of case* | | | | |
| *2019* | 36 (19) | 8 (10.1) | 1.00 | 1.00 |
| *2020* | 85 (45) | 35 (44.3) | 1.60 (0.81-3.19) 0.17 | 1.34 (0.61-3.03) 0.45 |
| *2021* | 68 (36) | 36 (45.6) | 1.90 (0.96-3.76) 0.06 | 1.63 (0.75-3.55) 0.21 |

| | | | | |
|---|---|---|---|---|
| *Antibiotic use before testing* | | | | |
| *No* | 72 (38.1) | 20 (25.3) | 1.00 | 1.00 |
| *Yes* | 117 (61.9) | 59 (74.7) | 1.54 (0.99-2.39) 0.05 | 1.28 (0.73-2.24) 0.38 |
| *Type of theatre* | | | | |
| *Others* | 182 (96.3) | 76 (96.2) | 1.00 | 1.00 |
| *Main theatre* | 7 (3.7) | 3 (3.8) | 1.02 (0.39-2.68) 0.97 | 0.91 (0.34-2.46) 0.855 |

# Discussion

This study provides the most recent epidemiology of SSTI and their resistance profiles in a Ugandan tertiary healthcare facility. The prevalence of SSTI was 66.4%, which was comparable to the prevalence reported in Pakistan (68.5%) (6), Sierra Leone (62.1%)(21) and Ethiopia (70%)(26). However, studies in the same settings including Mbarara RRH (81.93%, 92%) (27, 28) and Mulago National Referral Hospital (85%)(29) observed higher levels of infection. Much lower levels of skin infections, 1.5% (22), 3.1-4.4% (45), and 10.3-15.6% (25), have also been reported. These differences likely resulted from variations in the methods used to diagnose the infections. The clinical diagnostic approach based on physical examination was applied in some studies (22, 45) compared to the laboratory detection approach by C&S used in this research and the previous similar studies(27, 29).

Polymicrobial growth (7.2%) was observed where cases had more than one aetiologic agent isolated. This has been observed in other studies (12, 31, 34). In this study, *Candida* species were involved in more polymicrobial infections compared to the cases solely by a fungus, indicating that an SSTI by a fungal organism is more likely to occur with an existing bacterial agent. A higher proportion of mixed infections (21.4%) has previously been observed compared to only fungal infections (5.8%) (46). Also, there are significant interactions between bacteria and fungi to form biofilms reported to complicate healing, especially in chronic wound infections (47). This calls for further utilization of appropriate diagnostics to detect the fungal infections to limit unnecessary and prolonged use of antibiotics especially among patients with chronic deep tissue infections.

In this study, sex was not associated with SSTI or MDR as similarly observed in Benin (18) but contrary to other studies where sex was significantly associated with a higher risk for infection(19, 21). The age groups most affected by SSTI were those between 19 and 35 years followed by those between 36 and 59 years with SSTI in the 19-59 group being more likely to be due to MDR pathogens than those less than 12 years. This contrasts previous studies that reported a higher likelihood of infection among those above 35 years (24, 48). This might be because this age group is the most active in life, prone to injuries and risks that increase their probability of acquiring MDR infections. Age has previously not been associated with MDR infections elsewhere (49).

The proportion of patients tested in the microbiology laboratory for the management of SSTI was found to be 14.1%, which was slightly less than the 23% observed in California, USA (50). Treatment records for outpatients were not available while only 11 of the study patients' records were found to have culture and sensitivity result reports in inpatient files. These low numbers could be due to the non-electronic system used for patients' records and poor communication between

attending clinicians and the laboratory. This increases chances for misdiagnosis and poor choice of antibiotics to manage cases.

Most of the isolated bacteria were Gram-negative (58.6%(119/203)) as similarly observed by other studies in which the Gram-negative accounted for most of the infections ranging from 72.9-91% (19, 21, 30). However, *S. aureus* was individually responsible for most of the infections observed in the current study. This is similar to studies conducted elsewhere (14, 31, 32). *S. aureus* was followed by *E. coli* then other bacteria such as *Coliforms*, *Klebsiella* species, *Citrobacter* species, and *Pseudomonas* species isolated. Similar organism rates have been reported in China, Ethiopia, and Rwanda (12, 22, 30).

This study observed that 47% of the infections were due to MDR pathogens, which are greater than the 22.6% prevalence observed in Poland (33). This difference could be due to the employment of better infection prevention measures compared to Ugandan settings. Other studies also reveal that a large number of the bacteria responsible for the SSTI are MDR (21, 28, 34).

Up to 61% of Gram-negative bacteria were resistant to third-generation cephalosporins hence the possible presence of ESBL producers which was similar to 59.2% in Sierra Leone (21). Meanwhile, 27% were non-susceptible to carbapenems which is higher than the 8.2% observed among the Enterobacterales (21). The study observed MRSA levels of 44% which is comparable to Ethiopia (49%) (26) but less than levels reported in Saudi Arabia(65.4%) (51). The observation was greater than what is reported in previous studies from Poland (23.6%) (33) and the average in Sub-Saharan Africa (23.9%) (2).

All the *S. aureus* isolates tested were resistant to penicillin G. However, gentamicin and ciprofloxacin had higher sensitivity compared to the other agents as similarly observed in Ethiopia

(26). *Enterobacterales* on the other hand showed the highest resistance to ampicillin. A similar observation was reported earlier (2). The current study shows that the best agents for managing infections due to *Enterobacterales* currently include imipenem, gentamicin, and chloramphenicol. Isolates of *Acinetobacter* species and *Pseudomonas* species together (n=18) had a percentage resistance of 55.6%, 50%, 50%, 36.4%, 25%, and 22.2% against piperacillin, amikacin, ceftazidime, ciprofloxacin, gentamicin, and imipenem respectively. However, their number was less than the threshold necessary to generate reliable antibiograms. Therefore, recommendations about their empirical management cannot be appropriately made based on the available information. Previously, piperacillin+tazobactam has been recommended for use against Pseudomonas aeruginosa (6).

Patients in the surgical ward were 1.78 times more likely to develop an infection compared to those in the accidents and emergency ward. The outpatient department also had a lower prevalence of infection compared to the surgical ward. This could be due to the possession of open wounds from surgical repair that increase their liability to acquiring infections. Other studies have significantly associated infection with patients with a history of surgery and admission unit(12, 18).

Patients with prior antibiotic exposure before microbiology testing were not likely to have an SSTI due to MDR aetiologic agent compared to those who were unexposed. A similar outcome was observed earlier (34). The factors that had positive associations with MDR infections included type of ward, type of theatre, gender of the patient, and year of case. However, these were not statistically significant.

This study had some limitations, including being based at a single facility and missing data for some variables. Out-patients had no treatment files available and some inpatient files could not be located due to the manual hardcopy filing system. This limited the ability to obtain information

such as surgical history, theatre involved, length of admission, and treatment records. These were recorded as unknown for some patients. Individual variables with insignificant data (Less than 30 observations) could not be concluded. The observed number of patients with culture and sensitivity results in their files could not be used to generate a representative proportion of de-escalation based on test results. Unknown data regarding some variables such as undergoing surgery, type of surgery, and the theatre could have affected their outcome as possible associated factors for SSTI and AMR. The private wing of the facility though on a small scale involves some specialized medical service units such as gynecology, and pediatrics, among others. However, no disaggregated data was readily available to individually analyze cases of their origin. Other conditions such as comorbidities, wound classes, surgical antimicrobial prophylaxis, and surgeons' experience were not assessed due to data shortage. There was no follow-up of patients to ascertain clinical outcomes post-treatment. This is encouraged for inclusion in future studies to provide a full picture and signify the relationship between practice, risk factors, and the final outcome for better management.

# Conclusions

The prevalence of SSTI was high at Jinja RRH, with only a few cases of SSTI being tested for culture and sensitivity. The Gram-negative were the most responsible pathogens for the SSTI while the most isolated individual pathogen was *S. aureus*. Almost half of the infections were due to MDR pathogens including MRSA, possible ESBL-producers, and organisms that are non-susceptible to carbapenems. Recognizing SSTI under the GLASS would lead to enhanced surveillance, better preparedness, and response to AMR.

# Author Contributions

**Conceptualization**, F.L.; **methodology**, F.L.; **data curation**, F.L.; **formal analysis**, F.L. I.N. J.B.K. and J.K.; **investigation**, D.K. M.O. F.K; **writing—original draft preparation**, F.L.; **writing—review and editing**, I.R.; J.K. S.D.K. J.M. D.K. D.T M.O. F.K. J.B.K. and I.N **visualization**, S.D.K. J.M. and I.R. All authors have read and agreed to the published version of the manuscript.

# Acknowledgments

The author appreciates Ms. Zainab Kirunda Kitimbo; Ms. Fauzia Namora; Ms. Sarah Kyoyagala and Ms. Hadia Mukyala for the support provided and reading for clarity.

# References

1. Kiggundu R, Wittenauer R, Waswa J, Nakambale HN, Kitutu FE, Murungi M, et al. Point prevalence survey of antibiotic use across 13 hospitals in Uganda. Antibiotics. 2022;11(2):199.
2. Lai PS, Bebell LM, Meney C, Valeri L, White MC. Epidemiology of antibiotic-resistant wound infections from six countries in Africa. BMJ global health. 2018;2(Suppl 4):e000475.
3. O'neill J. Antimicrobial resistance: tackling a crisis for the health and wealth of nations. Rev Antimicrob Resist. 2014.
4. Mestrovic T, Aguilar GR, Swetschinski LR, Ikuta KS, Gray AP, Weaver ND, et al. The burden of bacterial antimicrobial resistance in the WHO European region in 2019: A cross-country systematic analysis. The Lancet Public Health. 2022;7(11):e897-e913.
5. Bonniface M, Nambatya W, Rajab K. An evaluation of antibiotic prescribing practices in a rural refugee settlement district in Uganda. Antibiotics. 2021;10(2):172.
6. Chaudhary NA, Munawar MD, Khan MT, Rehan K, Sadiq A, Bhatti HW, et al. Epidemiology, bacteriological profile, and antibiotic sensitivity pattern of burn wounds in the burn unit of a tertiary care hospital. Cureus. 2019;11(6).
7. Okello N, Oloro J, Kyakwera C, Kumbakumba E, Obua C. Antibiotic prescription practices among prescribers for children under five at public health centers III and IV in Mbarara district. PLoS One. 2020;15(12):e0243868.
8. Peetermans M, de Prost N, Eckmann C, Norrby-Teglund A, Skrede S, De Waele J. Necrotizing skin and soft-tissue infections in the intensive care unit. Clinical Microbiology and Infection. 2020;26(1):8-17.
9. Sartelli M, Guirao X, Hardcastle TC, Kluger Y, Boermeester M, Raşa K, et al. 2018 WSES/SIS-E consensus conference: recommendations for the management of skin and soft-tissue infections. World Journal of Emergency Surgery. 2018;13(1):1-24.
10. Uganda Go. Uganda National National Action Plan for Antimicrobial Resistance 2018-2023 [cited 2023 29/11/2023]. Available from: https://www.cphl.go.ug/sites/default/files/2020-

02/Uganda%20National%20Action%20Plan%20for%20Antimicrobial%20Resistance%202018-%202023-compressed_0.pdf.

11. Alamrew K, Tadesse TA, Abiye AA, Shibeshi W. Surgical antimicrobial prophylaxis and incidence of surgical site infections at Ethiopian Tertiary-Care Teaching Hospital. Infectious Diseases: Research and Treatment. 2019;12:1178633719892267.

12. Alemayehu T. The burden of aerobic bacterial nosocomial infections, associated risk factors and antibiotic susceptibility patterns in a surgical site in Ethiopia: A systematic review. J Surg Surgical Res. 2020;6(2):126-32.

13. Kefale B, Tegegne GT, Degu A, Molla M, Kefale Y. Surgical site infections and prophylaxis antibiotic use in the surgical ward of public hospital in Western Ethiopia: a hospital-based retrospective cross-sectional study. Infection and Drug Resistance. 2020:3627-35.

14. Morgan E, Hohmann S, Ridgway JP, Daum RS, David MZ. Decreasing incidence of skin and soft-tissue infections in 86 US emergency departments, 2009–2014. Clinical Infectious Diseases. 2019;68(3):453-9.

15. Mancuso G, Midiri A, Gerace E, Biondo C. Bacterial Antibiotic Resistance: The Most Critical Pathogens. Pathogens. 2021;10(10).

16. Salamzade R, Manson AL, Walker BJ, Brennan-Krohn T, Worby CJ, Ma P, et al. Inter-species geographic signatures for tracing horizontal gene transfer and long-term persistence of carbapenem resistance. Genome Medicine. 2022;14(1):37.

17. Chaoui L, Mhand R, Mellouki F, Rhallabi N. Contamination of the surfaces of a health care environment by multidrug-resistant (MDR) bacteria. International journal of microbiology. 2019;2019.

18. Dégbey C, Kpozehouen A, Coulibaly D, Chigblo P, Avakoudjo J, Ouendo E-M, et al. Prevalence and Factors Associated With Surgical Site Infections in the University Clinics of Traumatology and Urology of the National University Hospital Centre Hubert Koutoukou Maga in Cotonou. Frontiers in Public Health. 2021;9:629351.

19. Wong KA, Holloway S. An observational study of the surgical site infection rate in a General Surgery Department at a General Hospital in Malaysia. Wounds Asia. 2019;2(2):10-9.

20. Alkaaki A, Al-Radi OO, Khoja A, Alnawawi A, Alnawawi A, Maghrabi A, et al. Surgical site infection following abdominal surgery: a prospective cohort study. Canadian journal of surgery. 2019;62(2):111.

21. Lakoh S, Yi L, Sevalie S, Guo X, Adekanmbi O, Smalle IO, et al. Incidence and risk factors of surgical site infections and related antibiotic resistance in Freetown, Sierra Leone: a prospective cohort study. Antimicrobial Resistance & Infection Control. 2022;11(1):1-12.

22. Wang B, Xiao X, Zhang J, Han W, Hersi SA, Tang X. Epidemiology and microbiology of fracture-related infection: a multicenter study in Northeast China. Journal of Orthopaedic Surgery and Research. 2021;16(1):1-11.

23. Liang Z, Rong K, Gu W, Yu X, Fang R, Deng Y, et al. Surgical site infection following elective orthopaedic surgeries in geriatric patients: incidence and associated risk factors. International Wound Journal. 2019;16(3):773-80.

24. Zejnullahu VA, Isjanovska R, Sejfija Z, Zejnullahu VA. Surgical site infections after cesarean sections at the University Clinical Center of Kosovo: rates, microbiological profile and risk factors. BMC infectious diseases. 2019;19(1):1-9.

25. Sway A, Nthumba P, Solomkin J, Tarchini G, Gibbs R, Ren Y, et al. Burden of surgical site infection following cesarean section in sub-Saharan Africa: a narrative review. International journal of women's health. 2019:309-18.

26. Sisay M, Worku T, Edessa D. Microbial epidemiology and antimicrobial resistance patterns of wound infection in Ethiopia: a meta-analysis of laboratory-based cross-sectional studies. BMC Pharmacology and Toxicology. 2019;20(1):1-19.

27. Wangoye K, Mwesigye J, Tungotyo M, Twinomujuni Samba S. Chronic wound isolates and their minimum inhibitory concentrations against third generation cephalosporins at a tertiary hospital in Uganda. Scientific Reports. 2022;12(1):1195.

28. Hope D, Ampaire L, Oyet C, Muwanguzi E, Twizerimana H, Apecu RO. Antimicrobial resistance in pathogenic aerobic bacteria causing surgical site infections in Mbarara regional referral hospital, Southwestern Uganda. Scientific reports. 2019;9(1):17299.

29. Wekesa YN, Namusoke F, Sekikubo M, Mango DW, Bwanga F. Ceftriaxone-and ceftazidime-resistant Klebsiella species, Escherichia coli, and methicillin-resistant Staphylococcus aureus dominate caesarean surgical site infections at Mulago Hospital, Kampala, Uganda. SAGE Open Medicine. 2020;8:2050312120970719.

30. Mukagendaneza MJ, Munyaneza E, Muhawenayo E, Nyirasebura D, Abahuje E, Nyirigira J, et al. Incidence, root causes, and outcomes of surgical site infections in a tertiary care hospital in Rwanda: a prospective observational cohort study. Patient Safety in Surgery. 2019;13:1-8.

31. Kaye KS, Petty LA, Shorr AF, Zilberberg MD. Current epidemiology, etiology, and burden of acute skin infections in the United States. Clinical Infectious Diseases. 2019;68(Supplement_3):S193-S9.

32. Pal S, Sayana A, Joshi A, Juyal D. Staphylococcus aureus: A predominant cause of surgical site infections in a rural healthcare setup of Uttarakhand. Journal of Family Medicine and Primary Care. 2019;8(11):3600.

33. Pawłowska I, Ziółkowski G, Wójkowska-Mach J, Bielecki T. Can surgical site infections be controlled through microbiological surveillance? A three-year laboratory-based surveillance at an orthopaedic unit, retrospective observatory study. International orthopaedics. 2019;43:2009-16.

34. Alfouzan W, Al Fadhli M, Abdo N, Alali W, Dhar R. Surgical site infection following cesarean section in a general hospital in Kuwait: trends and risk factors. Epidemiology & Infection. 2019;147:e287.

35. GLASS. GLASS manual for antimicrobial resistance surveillance in common bacteria causing human

infection. 2023 [cited 2023 29/11/2023]. Available from: https://iris.who.int/bitstream/handle/10665/372741/9789240076600-eng.pdf?sequence=1&isAllowed=y.

36. Denis Edema JN. Six dead, 11 injured as cement truck rams into taxi along Jinja-Kampala highway: Monitor; 2022 [Available from: https://www.monitor.co.ug/uganda/news/national/six-dead-11-injured-as-cement-truck-rams-into-taxi-along-jinja-kampala-highway-4045636.

37. Onyango J. One critically injured in accident involving 3 vehicles: NTV; 2023 [Available from: https://www.ntv.co.ug/ug/news/national/one-critically-injured-in-accident-involving-3-vehicles-4136380.

38. Richard N. Fatal Jinja Road Accident: Next media; 2021 [Available from: https://nbs.ug/2021/12/fatal-jinja-road-accident/.

39. Jinja-RRH. About Jinja Regional Referral Hospital 2022 [cited 2023 09/09/2023]. Available from: https://jinjahospital.go.ug/about-us/

40. SANAS. Accredited organizations/Medical Laboratories/Jinja Regional Referral Hospital Laboratory 2021 [cited 2023 09/09/2023]. Available from: https://www.sanas.co.za/Pages/index.aspx

41. CLSI. Performance Standards for Antimicrobial Susceptibility Testing.28th ed. CLSI guideline M100. Wayne, PA: Clinical and Laboratory Standards Institute 2018.
42. CLSI. Performance Standards for Antimicrobial Susceptibility Testing.30th ed. CLSI guideline M100. Wayne, PA: Clinical and Laboratory Standards Institute. 2020.
43. CLSI. Performance Standards for Antimicrobial Susceptibility Testing.31st ed. CLSI guideline M100. Wayne, PA: Clinical and Laboratory Standards Institute. 2021.
44. Magiorakos AP, Srinivasan A, Carey RB, Carmeli Y, Falagas ME, Giske CG, et al. Multidrug-resistant, extensively drug-resistant and pandrug-resistant bacteria: an international expert proposal for interim standard definitions for acquired resistance. Clin Microbiol Infect. 2012;18(3):268-81.
45. Kizito O. Comparative study of proportions of post-operative sepsis _ maternity versus general surgical ward. Cogent Medicine. 2021;8(1):1889100.
46. Chellan G, Shivaprakash S, Karimassery Ramaiyar S, Varma AK, Varma N, Thekkeparambil Sukumaran M, et al. Spectrum and prevalence of fungi infecting deep tissues of lower-limb wounds in patients with type 2 diabetes. Journal of clinical microbiology. 2010;48(6):2097-102.
47. Kalan L, Grice EA. Fungi in the Wound Microbiome. Adv Wound Care (New Rochelle). 2018;7(7):247-55.
48. Yousif SM, Abakar AD, Nour BY, Ibrahim SO, Elhasan OMA, Yousif MA, et al. Frequency and antimicrobials susceptibility pattern of Staphylococcus aureus associated with wound infections in surgery department, wad madani teaching hospital, sudan. Pharmacology & Pharmacy. 2021;12(12):334-43.
49. Alsehemi AF, Alharbi EA, Alammash BB, Alrais AI, Elbadawy HM, Alahmadi YM. Assessment of risk factors associated with multidrug-resistant organism infections among patients admitted in a tertiary hospital - a retrospective study. Saudi Pharmaceutical Journal. 2023;31(6):1084-93.
50. Ray GT, Suaya JA, Baxter R. Incidence, microbiology, and patient characteristics of skin and soft-tissue infections in a U.S. population: a retrospective population-based study. BMC Infectious Diseases. 2013;13(1):252.
51. Almuhayawi MS, Alruhaili MH, Gattan HS, Alharbi MT, Nagshabandi M, Al Jaouni S, et al. Staphylococcus aureus Induced Wound Infections Which Antimicrobial Resistance, Methicillin- and Vancomycin-Resistant: Assessment of Emergence and Cross Sectional Study. Infect Drug Resist. 2023;16:5335-46.

Click here to access/download;Figure;Fig1.tif

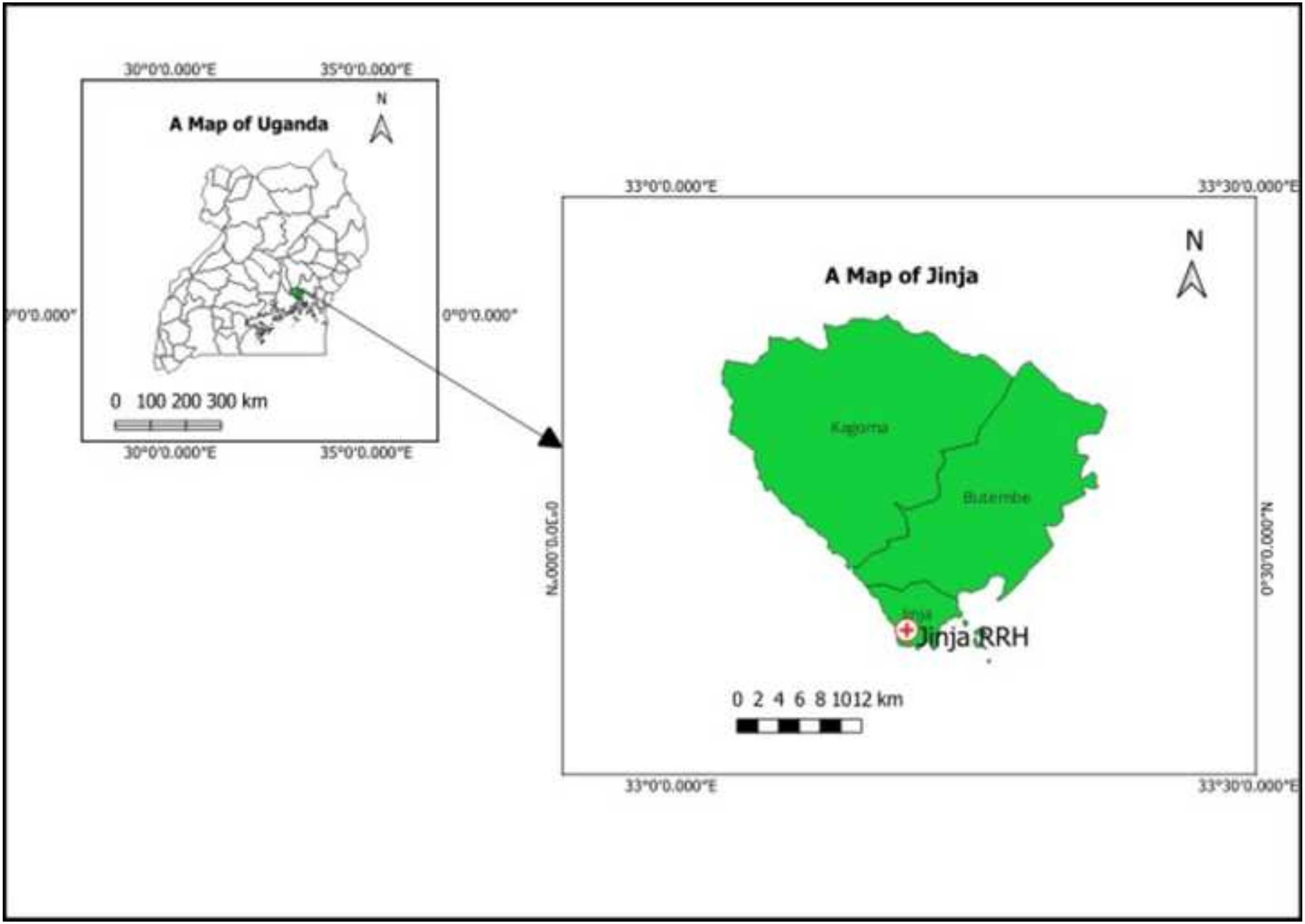

Click here to access/download;Figure;Fig2.tif

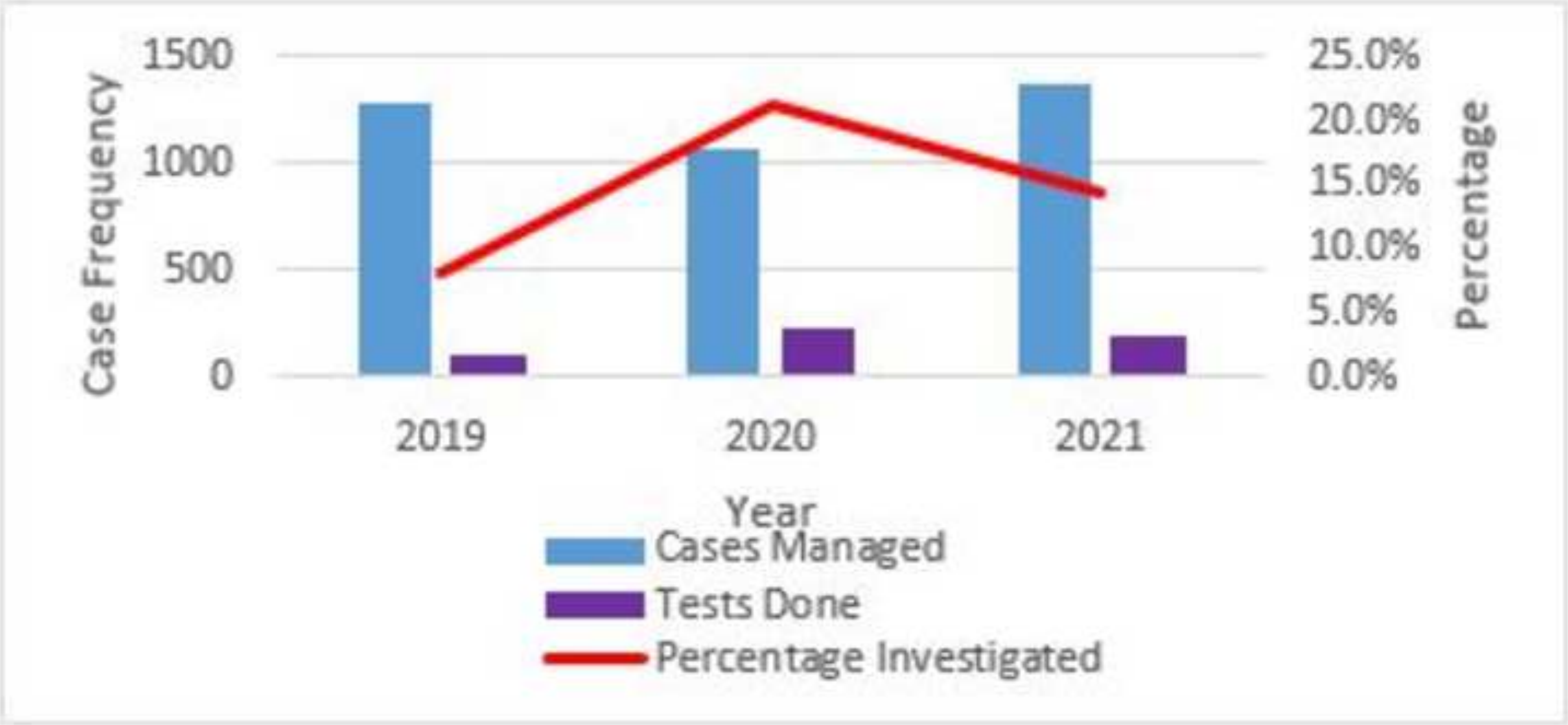

Click here to access/download;Figure;Fig3.tif

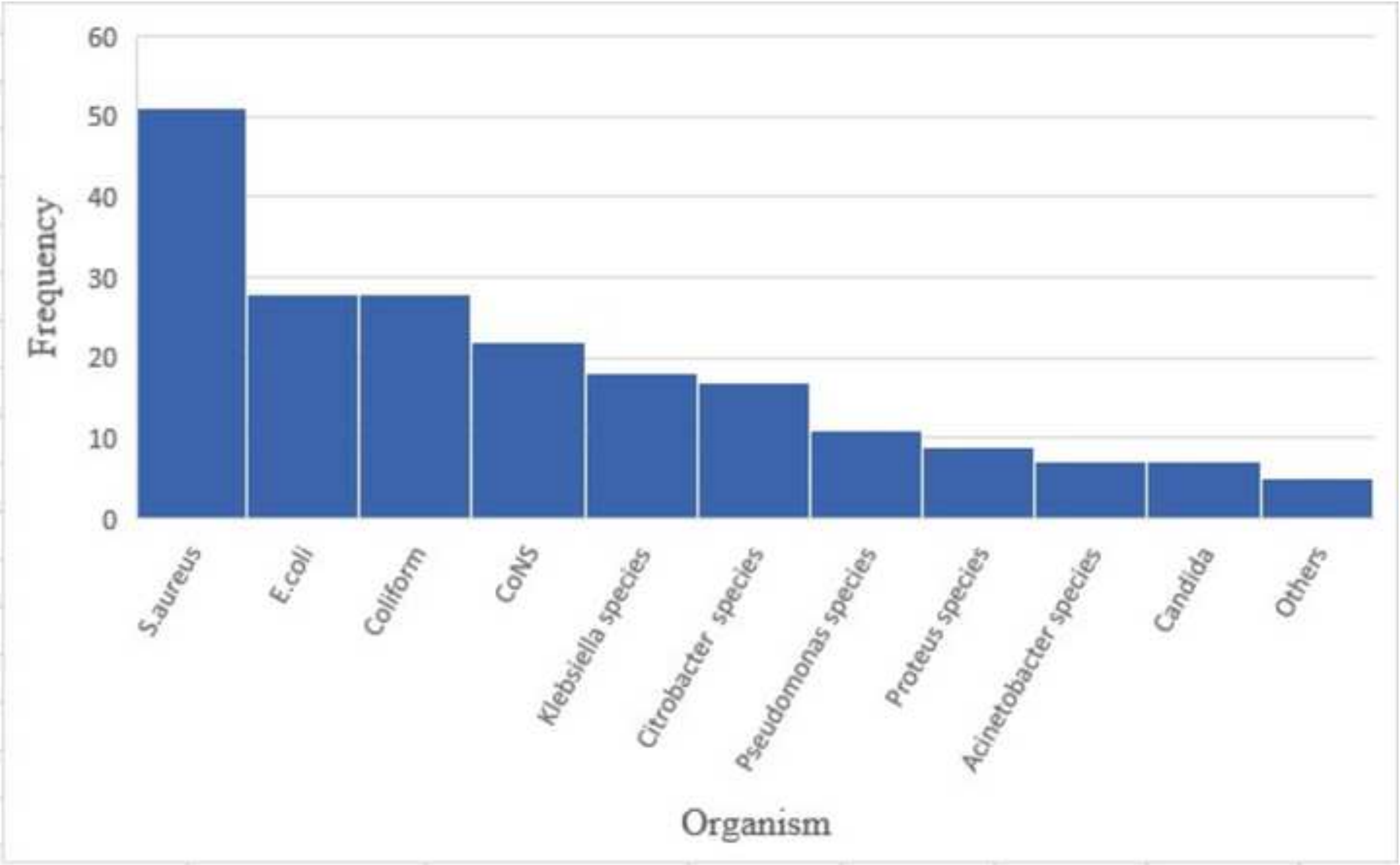

Click here to access/download;Figure;Fig4.tif

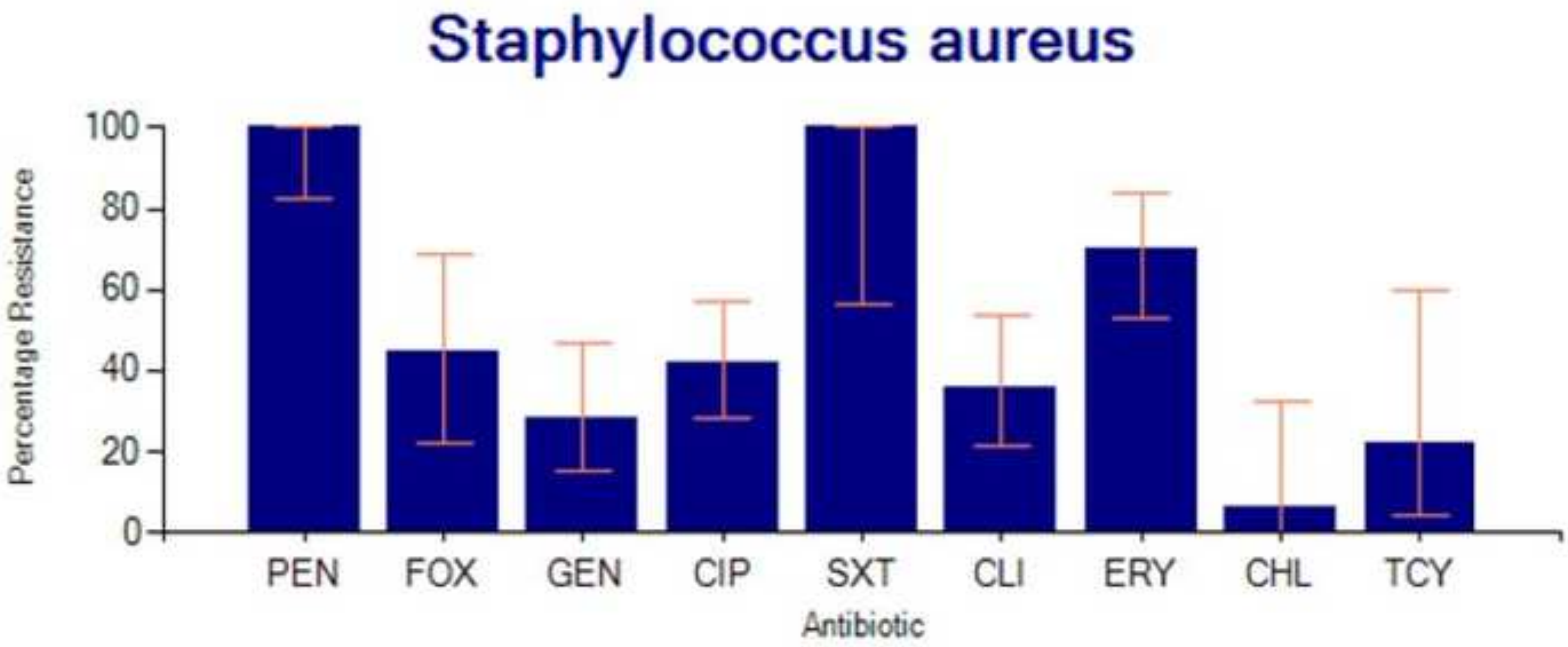


PEN-Penicillin G; FOX-Cefoxitin; GEN-Gentamicin; CIP-Ciprofloxacin; SXT-Co-trimoxazole; CLI-Clindamycin; ERY-Erythromycin; CHL-Chloramphenicol; TCY-Tetracycline

Click here to access/download;Figure;Fig5.tif

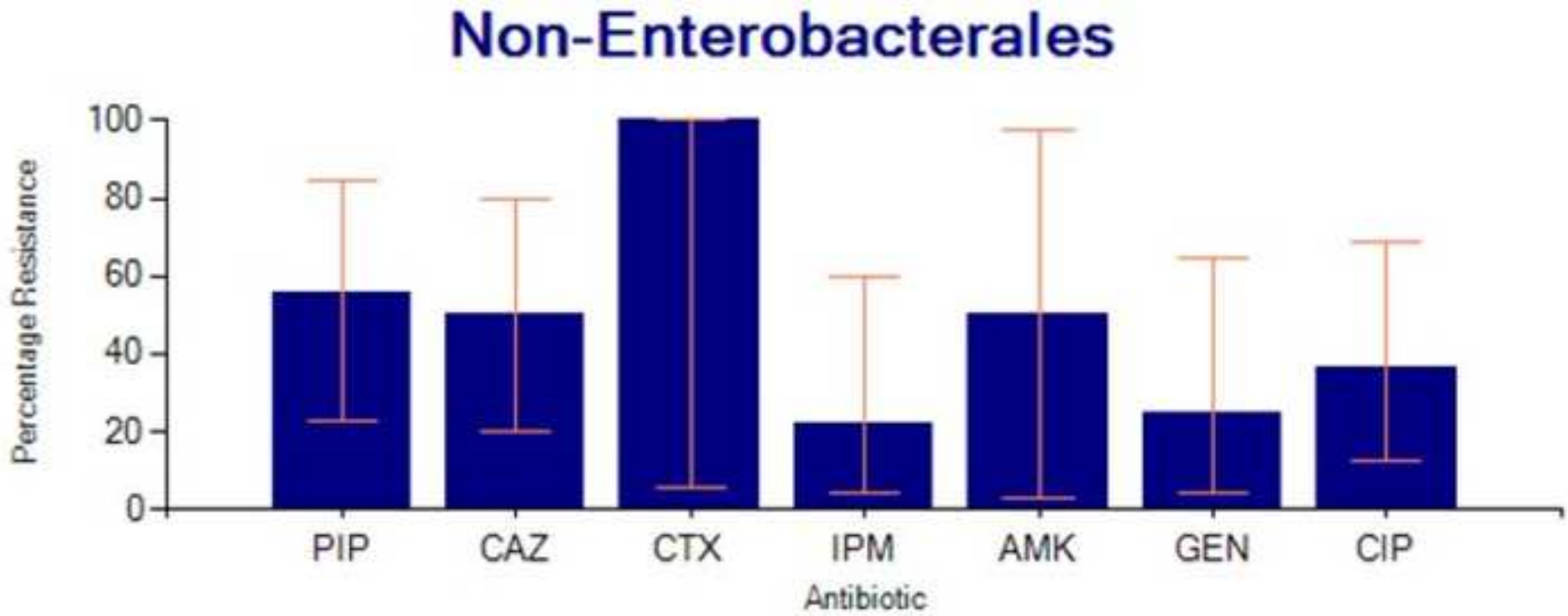


PIP-Piperacillin; CAZ-Ceftazidime; CTX-Cefotaxime; IPM-Imipenem; AMK-Amikacin; GEN-Gentamicin; CIP-Ciprofloxacin
