# Supplementary material for: Prevalence, resistance profiles and factors associated with skin and soft-tissue infections at Jinja regional referral hospital: A retrospective study
Source: PLOS Glob Public Health. 2024 Aug 2;4(8):e0003582. doi: 10.1371/journal.pgph.0003582 (PMC11296629; doi:10.1371/journal.pgph.0003582)
Supplement: Attachment — Submitted filename: Response to Reviewers.docx [file pgph.0003582.s003.docx]

26/06/2024

Academic Editor
PLOS Global Public Health

Dear Dr. Mosi,

We greatly appreciate the revision and comments provided on the manuscript PGPH-D-24-00292 titled “Prevalence, resistance profiles and factors associated with skin and soft-tissue infections at Jinja regional referral hospital: A retrospective Study”

Below are the responses to the respective comments.

**Journal Requirements:**

1. *Please provide additional details regarding participant consent. If you are reporting a retrospective study of medical records or archived samples, please ensure that you have discussed whether all data were fully anonymized before you accessed them and/or whether the IRB or ethics committee waived the requirement for informed consent. If patients provided informed written consent to have data from their medical records used in research, please include this information.*

**Response:** Thank you for this comment. The ethics statement has been re-written and currently reads as “The Jinja Hospital Research and Ethics Committee (JREC) approved the study with registration number **JREC 395/2023.** The JREC granted a waiver for informed consent and medical records were anonymized prior to analysis for this study.” Kindly see line 154-156 of the revised protocol. Also attached is the letter from the institutional review board.

*2. We have noticed that you have uploaded Supporting Information files, but you have not included a list of legends. Please add a full list of legends for your Supporting Information files after the references list.*

**Response:** The supporting information legend has been added for the Research and Ethics Committee Approval and Waiver Letter as required. Please see lines; 550-555.

*3. Some material included in your submission may be copyrighted. According to PLOS’s copyright policy, authors whouse fi2gures or other material (e.g., graphics, clipart, maps) from another author or copyright holder must demonstrate or obtain permission to publish this material under the Creative Commons Attribution 4.0 International (CC BY 4.0)License used by PLOS journals. Please closely review the details of PLOS’s copyright requirements here: PLOSLicenses and Copyright. If you need to request permissions from a copyright holder, you may use PLOS's CopyrightContent Permission form.*

# Response: Much appreciation for this comment. Information about the source of the shape file for the map and the license information has been added to the legend of Fig 1 and currently reading as “Fig 1. A map showing the location of the study site. This map was created using QGIS Desktop 3.32.1. The base layer is freely accessible from <https://diva-gis.org/data>. This can be shared under CC-BY license 4.0.” Please see lines; 109-111 in the revised manuscript.

*4. Please note: the data from the URL you have provided is not available. Please ensure your data is made publicly available so that we may proceed with production.*

## **Response:** The dataset for the study was uploaded on a public site (https://datadryad.org/stash/dashboard) and currently under review by the site team for publication. Also, site handlers assure that it is made public as soon as the manuscript is accepted by the journal. The data is currently accessible via <https://datadryad.org/stash/share/-AnX_6jNPSHehz4OQAscWsUbr0rDnfQk_2aPARLuGoU>. The permanent access link after review will remain <https://doi.org/10.5061/dryad.rjdfn2zkh>.

**Major editorial comments:**

**Reviewer #1:**

***Comment 1:*** *Abstract: It should be structured*

**Response:** Thank you for this comment. However, the journal instructions to authors required using a non-structured abstract.

***Comment 2:Briefly explain the gap why you study? Plausible and rigorously justify the gap what information limited here***

**Response:** This comment was referred to line 87-89 in the introduction. This section has been revised to expound the problem statement and filled study gap. Please see lines 70-81 in the revised manuscript.

***Comment 3:*** Methods – *is retrospective study included all variable in the registration that the investigators objective answered?*
**Response:** We believe you meant whether all variables were investigated in a retrospective nature. This is true as the study objectives were achieved based on existing records from the laboratory and patients’ files. This was the approach used for data collection as mentioned in the methods section. Please see lines 122-152 of the updated protocol.

***Comment 3:*** *What do you if registration chart is incomplete?*
**Response:** Any variable whose observations were incomplete, the missing proportion was noted as unknown. Please see Table 1. Those with less than thirty observations were omitted from analysis such as the comorbidities including HIV Status and percentage of treatment de-escalation based on the culture and sensitivity report. Please see lines 208-210 in the updated protocol.

***Comment 4:*** *Is the registration book or chart standard for all required information?*

**Response:** Two standard systems were the data sources for the study and extraction from both was described in the methods. This includes the laboratory information system (Lines 121-126) and standard patient files (HMIS 108 and HMIS 105:01).

***Comment 5:*** *Analysis methods: - what is your analysis model?*
**Response:** The model used to assess association among variables was the modified Poisson regression. Please see lines 177-178.

***Comment 6:*** *Model fitness*
**Response:** More information has been included about fitting the model. Please see lines 177-179 in the updated manuscript.

***Comment 7:*** *VIF( variable inflation factors)*

**Response:** The Variance Inflation Factors (VIF) for the predictor variables were all less than 10; CaseYear-1.33, Ward-1.13, Sex-1.03, Age-1.03, Admission48Hrs-2.12, surgery-1.44, surgical type-2.15, Antibiotics-1.95, theatre-2.07. The Mean VIF has been added to the methods section. Please see lines 174-175 in the updated manuscript.

***Comment 8:*** *Results : what is your response rate?*
**Response:** All records equivalent to the sample size necessary for determining the overall prevalence and resistance profiles were accessed. Please see line 183 in the updated protocol. However, some variables had missing data such as patients that underwent surgery and the missing proportion was reported as unknown. Please see Table 1.

***Comment 9:*** *Conclusion: is your conclusion rigorous or strong based your finding?*

**Response:** The conclusion has been modified. Please see lines 38-40 in the new manuscript.

**Reviewer #2:**

*The manuscript titled: “Prevalence, resistance profiles and factors associated with skin and soft-tissue infections at Jinja regional referral hospital: A retrospective Study” addresses an important topic related to the challenges associated with AMR in the healthcare space. The authors find a considerable degree of AMR among patients with wound infections. The authors examine MDR and its associated factors and present a case for a specific set of antimicrobials for the treatment of wound infections. The authors should be applauded for the job well done in general. I have just a few comments: -*
***Comment 1:*** *Line 48-50 reads “Using microbiology services such as culture and sensitivity (C&S) testing enables determination of the identity of the causative agents and the appropriate antimicrobial agents”. This can be made clearer by cutting down unnecessary words eg “determination or the causative agent…”*

**Response:** The sentence has been improved and currently reading as “This involves microbiology services such as culture and sensitivity (C&S) testing to enable identification of causative agents and the appropriate antimicrobial agents for managing individuals with infections…..” Please see lines 53-56 in the updated manuscript.

***Comment 2:*** *Line 52: “However, this this is still far from the practice in this setting as most clinical management is still” remove the extra word “this”*

**Response:** The sentence in the current line 56-57 has been modified and currently reading “…..there is still a low coverage in this setting….”

***Comment 3:*** *Line 128-130: reads “The sample size was calculated using the formula (n =p(1-p)z2/d2) and had a power of 80% to estimate the prevalence and factors associated with SSTI at the 95% confidence level.”*
***Comment 4:*** *We need more information on the `prevalence’ and `d’ to be able to replicate the sample size*.

**Response:** The prevalence ‘p’ and precision ‘d’ have been added. Please see lines 117-118 in the updated protocol.

***Comment 5:*** *Line 183 - 186 Bivariate and Multivariate terminologies are used. Please change them to Univariable and multivariable analyses.*

**Response:** The terms have been renamed as recommended. Please see lines 175-178 in the updated protocol.

***Comment 6:*** *I would combine table 1 and table 2. There is no need to have them separates as they are. The rows should have the various social demographic and clinical factors while the column should have total, positive and percentage.*

**Response:** The tables have been combined as suggested. Please see Table 1 in the updated protocol.

***Comment 7:*** *Some sentences need rewriting eg “In 2019, there were 8.1% (104/1278) cases that underwent laboratory testing for SSTI. Meanwhile, years 2020 and 2021 respectively had 21.1% (226/1072) and 14.3% (196/1370) cases diagnosed by the laboratory to guide management (Fig 2).”*

**Response:** The sentence has been modified into “In 2019, only 8.1% (104/1278) of suspected SSTI cases underwent laboratory confirmation while 21.1% (226/1072) and 14.3% (196/1370) were laboratory confirmed in 2020 and 2021 respectively. (**Fig 2**).” Please see lines 205-207 of the updated manuscript.

***Comment 8:*** *The figure and table legends need more information to guide the reader.*

**Response:** More information has been added to the figure and table legends in the updated manuscript.

***Comment 9:*** *Line 261: “d (aPR = 1.78, 95%, CI:1.003-3.18, p = 0.04).” ensure consistency in decimal places.*
**Response:** The decimal places (dp) have been updated and currently used categorically; 0 dp(General whole numbers), 1dp(General percentages), 2dp(CIs, PRs and P-values). Please see Tables; 2, 3, 4 & 5 in the updated manuscript.

***Comment 10:*** *Table 5: change bivariate and multivariate to univariable and multivariable*
**Response:** The terms have been renamed as recommended. Please see Table 4 and 5 in the revised manuscript.

***Comment 11:*** *The authors found that age was associated with MDR, however, besides contrasting /comparing with previous studies from elsewhere, there is need to provide a substantive explanation/hypothesis on why that is the case. I would expect that children may be exposed to health facilities more and therefore more likely to demonstrate resistance (MDR) compared adults. However, this does not seem to be the case. Did you assess length of hospital stay, HIV status (as a proxy for immunity), wound severity, History of hospital admissions etc. These variables would likely confound age.*

**Response:** A possible explanation for the observation has been included in the discussion stating “This might be because this age group is the most active in life, prone to injuries and therefore exposure to antibiotics during treatment of the injuries, increasing their risk for MDR infections. Additionally, these are more likely to access antibiotics through self-medication, which increases the risk of AMR.” Please see lines 307-309 in the revised manuscript.

Length of hospital stay was assessed through two categories; those hospitalized for 48 hours or less and those hospitalized for longer than 48 hours. There was no statistically significant association found between the hospital admission history and MDR (Please see Table 5). There was insufficient data about HIV status and wound severity which limited their analysis.

*Reviewer #3:*

***Comment 1:*** *The scientific report was stressed in the paper, which was also presented. It will be approved after a incorporatent change if the author takes into account all of the comments made in the documents. The feedback provided by the reviewers highlighted areas for improvement, particularly in terms of data analysis and interpretation. Addressing these suggestions will greatly enhance the overall quality and impact of the research findings. Additionally, the reviewers suggested expanding the discussion section to provide more context and relevance to the results. Incorporating this feedback will strengthen the paper and make it more compelling for readers in the scientific community.*

**Response:** All the reviewers’ comments have been reasonably addressed to the best of our abilities.

**General Comments**

1. *It is better if you add few points as general background.*

**Response:** This comment was made to the abstract section. Extra points have been added to the abstract background as suggested. Please see lines 21-23 in the revised manuscript.

2. *Don’t personalize it, edit it*

**Response:** This comment was made to the abstract section for the word “We”. The sentence has been edited and currently starts as “This study...” Please see line 24 in the updated manuscript.

3. Line 23-24: *Re phrased it*

**Response:** The sentence has been rephrased into “This was based on records of patients suspected of SSTI for the period of 2019-2021 at Jinja Regional Referral Hospital.” Please see line 25-27.

4. Line 26-27: *not clear, briefly state the general prevalence rate.*

**Response:** The general prevalence is directly stated as “The prevalence of SSTI was 66.4%.” Please see line 28 in the revised manuscript.

5. *Add the potential key words*

**Response:** Keywords have been added. Please see lines 42-43 in the revised manuscript.

6. *You introduce it well, but as you made complex introduction, thus why you failed to emphasis or come to your target. Please, reduce it for the maximum of 1 and half page, focus only your topic and your emphasized*

**Response:** The introduction has been modified and reduced as recommended. Please see lines 44-85 in the revised manuscript.

7. *For which organism did you use this procedures, because your data was already recorded data. I am not clear with this procedures make it clear.* (Line 115-121: Laboratory procedures)

**Response:** The title of ‘Laboratory procedures’ has been removed and the statement rephrased into “The laboratory results in this study were abstracted from the laboratory records. They were generated using the conventional microbiology methods for bacterial identification. Antimicrobial Susceptibility Testing(AST) was done using the Kirby-Bauer disk-diffusion method and interpreted according to the Clinical and Laboratory Standards Institute (CLSI) guidelines.” Please see lines 102-106 in the updated manuscript.

8. *Please make the correct procedures for investigate for all tests*

**Response:** This has been clarified as above. Please see lines 102-106 in the updated manuscript.

9. Line 132-137: *Is antimicrobial susceptibility test is extracted from data or experimental results. please, justify the procedures or put your justification.*

**Response:** The antimicrobial susceptibility testing (AST) results were part of the extracted data from laboratory records. The statement under laboratory procedures has been rephrased to clearly describe the routine methods originally used to generate the data used for this study. Please see lines 102-106 and 122-126 in the updated manuscript.

10. *Hoe did you calculate the prevalence rate, list and elaborate the correct calculation formula for the prevalence rate. Because, the prevalence rate in our study was high, then you need to justify the formula you used or the reason for high prevalence rate recorded.*

**Response:** The mode of calculating the prevalence has been clarified. Please see lines 161-163 in the updated manuscript.

11. *what the number ????????/ is the total, or why you add there, remove it or unless justify*

**Response:** This comment was related to line 198. The number has been clarified as the total positive cases based on to calculate the prevalence. The rephrased statement now reads as “The prevalence of SSTI was 66.4% (95% CI = 60.7-72.1) based on the 178 laboratory confirmed positive cases.” Please see lines 195-196 in the updated manuscript.

12. *The sources of bacteria, please specify the sources of bacteria, if isolated, specify the correct procedures or appropriate sources of bacteria or put your justification.*

**Response:** This has been clarified in the methods section describing the mechanisms by which the analyzed records were earlier generated (See lines: 102-106). And the data collection methods of how the records were obtained for the purpose of this study. Please see lines: 121-126 in the revised manuscript.

13. *What you mean ?*

**Response:** This comment was related to statement one of line 220. The sentence has been rephrased and now reads as “There were 203 organisms isolated from the clinical samples.” Please see line 217 in the revised manuscript.

14. *line 221 and is not clear incomplete, make it clear*

**Response:** The statements have been modified with proportions of organisms now reported relative to the total number of isolates. Please see lines: 217-222 in the updated manuscript.

15. *Do you think coliforms are pathogens or indicators, so , you need to explain and justify this issue, or specif which bacteria.*

**Response:** Only specific bacteria are reported in the revised manuscript. Please see lines: 217-221 in the updated manuscript.

16. *Line 233, first sentences is not clear,*

**Response:** The sentence has been rephrased and currently reading as “Close to 47% (79/171) of the isolated bacteria were MDR pathogens.” Please see line 227 in the updated protocol.

17. *concentration of drugs ....................for each*

**Response:** The drug concentrations of tested antibiotics have been added. Please see Table 2 and 3 in the revised manuscript.

18. *How this can be true, what about other possible factors like climate, topography, other conditions.*

**Response:** Thank you so much for this comment in relation to line 281. More possible causes for the observed difference in prevalence have been added. Please see lines 290-291 in the revised manuscript.

19. *line 285, is not written in good explaination*

**Response:** The sentence has been rephrased and currently reads as “Positive cases in which more than one aetiological agent was isolated (Polymicrobial growth) were 6.7%.” Please see lines 292-293 in the updated protocol.

20. *why the reason?*

**Response:** This comment was made on line 288. A possible explanation has been added. Please see line 295-296 in the updated manuscript.

21. Line 293: *Citation*

**Response:** A citation has been added to the explanation. Please see line 299.

22. *Avoid repeating the same idea* (Line 326)

**Response:** The extra comparison has been removed.

23. *at each point, justify the reason why they showed resistance to those drugs*

**Response:** Possible explanations for the observed resistance have been added. Please see lines 336-338 in the revised manuscript.

24. *Put strong recommendation* (Line 372)

**Response:** The conclusion has been modified to include extra recommendation. Please see lines 383-390 in the revised manuscript.
